# Academic Underachievement in Children with Active Epilepsy

**DOI:** 10.15844/pedneurbriefs-29-2-6

**Published:** 2015-02

**Authors:** Frank A. Zelko

**Affiliations:** 1 Department of Child and Adolescent Psychiatry, Ann & Robert H. Lurie Children's Hospital of Chicago; 2 Department of Psychiatry and Behavioral Sciences, Northwestern University Feinberg School of Medicine, Chicago, IL

**Keywords:** Cognition, Comprehension, Epilepsy

## Abstract

Investigators from Lingfield, Surrey, UK; University of Gothenburg, Sweden; Great Ormond Street Hospital, London, UK, and other centers, conducted psychological assessment including measures of IQ, working memory and processing speed in 85 (74%) of 115 school aged children with active epilepsy (a seizure in the past year and/or on AEDs) from a population-based sample, without exclusion for intellectual deficiency.

Investigators from Lingfield, Surrey, UK; University of Gothenburg, Sweden; Great Ormond Street Hospital, London, UK, and other centers, conducted psychological assessment including measures of IQ, working memory and processing speed in 85 (74%) of 115 school aged children with active epilepsy (a seizure in the past year and/or on AEDs) from a population-based sample, without exclusion for intellectual deficiency. Sixty-five (72%) of the 85 children who were able to complete subtests on the Wide Range Achievement Test Fourth Edition (WRAT-4) displayed low achievement (1 SD below mean), and 42% underachieved (1 SD below assessed IQ). Mean scores on the Math Computation subtest and Sentence Comprehension subtest were significantly lower than scores on Word Reading and Spelling subtests. Younger age at seizure onset was associated (p<0.05) with decreased scores on 3 of the 4 WRAT-4 subtests. Difficulties with auditory working memory were associated with difficulties on reading comprehension (p<0.05). Parent-reported difficulties with school attendance were associated with decreased scores on the Spelling and Word Reading subtests after controlling for IQ (p<0.05). The common occurrence of academic underachievement in school-aged children with “active” epilepsy can be attributed to lowered global cognition, specific cognitive deficits, younger onset of first seizure, and school attendance difficulties. Screening of school-aged children with “active epilepsy” for difficulties in school achievement should include identification of contributory factors and effective interventions. [1]

COMMENTARY. It is well established that children with epilepsy experience adverse academic outcomes [2, 3], but factors related to their academic difficulties are not fully understood. This study used a population-based approach to investigate academic achievement, diverging from past research in its failure to exclude, a priori, children with intellectual deficiency. As a result, the range of cognitive functioning sampled was broad and more representative of the full spectrum of childhood epilepsy. However, due to cognitive limitations 20 of the 85 participants were unable to complete items on the WRAT-4 screen, effectively restricting the range of analyses.

This study supports previous findings of adverse academic outcomes and indicates that deficits of processing speed and working memory confer particular risk for academic difficulties, with impact that appears greater in some areas (reading comprehension, math computation) than in others (reading decoding, spelling). It highlights the importance of early screening and close monitoring for academic difficulties in children with epilepsy, and underscores the need to consider contributing factors such as early age of seizure onset, school attendance difficulties, and lower overall cognitive functioning. However, academic difficulties often predate epilepsy diagnosis [2–4], suggesting that they may not be fully attributable to the process of epilepsy per se but instead associated with neurophysiologic factors that also give rise to epilepsy. Future studies should consider longitudinal approaches, a wider range of factors that could contribute to academic underachievement (e.g., child/parent/teacher attitudes), and a more comprehensive approach to cognitive assessment (including measures of attention, information processing efficiency, and executive functions).

## References

[1] Reilly C, Atkinson P, Das KB, Chin RF, Aylett SE, Burch V (2014). Academic achievement in school-aged children with active epilepsy: A population-based study. Epilepsia.

[2] Hermann B, Jones J, Sheth R, Dow C, Koehn M, Seidenberg M (2006). Children with new-onset epilepsy: neuropsychological status and brain structure. Brain.

[3] Oostrom KJ, Smeets-Schouten A, Kruitwagen CL, Peters AC, Jennekens-Schinkel A (2003). Not only a matter of epilepsy: early problems of cognition and behavior in children with “epilepsy only”--a prospective, longitudinal, controlled study starting at diagnosis. Pediatrics.

[4] Berg AT, Hesdorffer DC, Zelko FA (2011). Special education participation in children with epilepsy: what does it reflect?. Epilepsy Behav..

